# NeuroSAFE PROOF: study protocol for a single-blinded, IDEAL stage 3, multi-centre, randomised controlled trial of NeuroSAFE robotic-assisted radical prostatectomy versus standard robotic-assisted radical prostatectomy in men with localized prostate cancer

**DOI:** 10.1186/s13063-022-06421-7

**Published:** 2022-07-22

**Authors:** Eoin Dinneen, Jack Grierson, Ricardo Almeida-Magana, Rosie Clow, Aiman Haider, Clare Allen, Daniel Heffernan-Ho, Alex Freeman, Tim Briggs, Senthil Nathan, Susan Mallett, Chris Brew-Graves, Nicola Muirhead, Norman R. Williams, Elena Pizzo, Raj Persad, Jon Aning, Lyndsey Johnson, Jon Oxley, Neil Oakley, Susan Morgan, Fawzia Tahir, Imran Ahmad, Lorenzo Dutto, Jonathan M. Salmond, Anand Kelkar, John Kelly, Greg Shaw

**Affiliations:** 1 Division of Surgery & Interventional Science, University College London, London, UK; 2 University College Hospital London, Department of Urology, Westmoreland Street Hospital, 6-18 Westmoreland Street, W1G 8PH London, UK; 3 University College Hospital London, Department of Histopathology, 235 Euston Road, Bristol, NW1 2BU UK; 4 Division of Medicine, University College London, Charles Bell House, 43-45 Foley Street, Sheffield, W1W 7JN UK; 5 Department of Applied Health Research, University College London, 1-19 Torrington Place, Glasgow, WC1E 7HB UK; 6 North Bristol Hospitals Trust, Department of Urology, Southmead Hospital, Southmead Lane, Westbury-on-Trym, Bristol, BS10 5NB UK; 7 North Bristol Hospitals Trust, Department of Histopathology, Southmead Hospital, Southmead Lane, Westbury-on-Trym, BS10 5NB Bristol, UK; 8 Sheffield Teaching Hospitals NHS Trust, Department of Urology, Royal Hallamshire Hospital, Glossop Road, S10 2JF UK; 9 Sheffield Teaching Hospitals NHS Trust, Department of Histopathology, Royal Hallamshire Hospital, Glossop Road, S10 2JF UK; 10 Glasgow & Clyde NHS Trust, Department of Urology, Queen Elizabeth Hospital, 1345 Govan Road, Glasgow, UK; 11 Glasgow & Clude NHS Trust, Department of Histopathology, Queen Elizabeth Hospital, 1345 Govan Road, Glasgow, UK; 12 Barking Havering & Redbridge University Hospitals Trust, Rom Valley Way, Romford, RM7 0AG UK

**Keywords:** NeuroSAFE, Nerve sparing, Frozen section, Potency, Robotic prostatectomy, Prostate cancer, Randomised controlled trial, Protocol

## Abstract

**Background:**

Robotic radical prostatectomy (RARP) is a first-line curative treatment option for localized prostate cancer. Postoperative erectile dysfunction and urinary incontinence are common associated adverse side effects that can negatively impact patients’ quality of life. Preserving the lateral neurovascular bundles (NS) during RARP improves functional outcomes. However, selecting men for NS may be difficult when there is concern about incurring in positive surgical margin (PSM) which in turn risks adverse oncological outcomes. The NeuroSAFE technique (intra-operative frozen section examination of the neurovascular structure adjacent prostate margin) can provide real-time pathological consult to promote optimal NS whilst avoiding PSM.

**Methods:**

NeuroSAFE PROOF is a single-blinded, multi-centre, randomised controlled trial (RCT) in which men are randomly allocated 1:1 to either NeuroSAFE RARP or standard RARP. Men electing for RARP as primary treatment, who are continent and have good baseline erectile function (EF), defined by International Index of Erectile Function (IIEF-5) score > 21, are eligible. NS in the intervention arm is guided by the NeuroSAFE technique. NS in the standard arm is based on standard of care, i.e. a pre-operative image-based planning meeting, patient-specific clinical information, and digital rectal examination. The primary outcome is assessment of EF at 12 months. The primary endpoint is the proportion of men who achieve IIEF-5 score ≥ 21. A sample size of 404 was calculated to give a power of 90% to detect a difference of 14% between groups based on a feasibility study. Oncological outcomes are continuously monitored by an independent Data Monitoring Committee. Key secondary outcomes include urinary continence at 3 months assessed by the international consultation on incontinence questionnaire, rate of biochemical recurrence, EF recovery at 24 months, and difference in quality of life.

**Discussion:**

NeuroSAFE PROOF is the first RCT of intra-operative frozen section during radical prostatectomy in the world. It is properly powered to evaluate a difference in the recovery of EF for men undergoing RARP assessed by patient-reported outcome measures. It will provide evidence to guide the use of the NeuroSAFE technique around the world.

**Trial registration:**

NCT03317990 (23 October 2017). Regional Ethics Committee; reference 17/LO/1978.

**Supplementary Information:**

The online version contains supplementary material available at 10.1186/s13063-022-06421-7.

## Administrative information

Note: the numbers in curly brackets in this protocol refer to SPIRIT checklist item numbers. The order of the items has been modified to group similar items (see http://www.equator-network.org/reporting-guidelines/spirit-2013-statement-defining-standard-protocol-items-for-clinical-trials/).

**Table:** 

Title {1}	NeuroSAFE PROOF: study protocol for a single-blinded, IDEAL stage 3, multi-centre, randomised controlled trial of NeuroSAFE robotic-assisted radical prostatectomy versus standard robotic-assisted radical prostatectomy in men with localized prostate cancer.
Trial registration {2a and 2b}.	NCT03317990 (23 October 2017). Regional Ethics Committee; reference 17/LO/1978.
Protocol version {3}	NeuroSAFE PROOF protocol version 5.0 was granted on 16 June 2021.
Funding {4}	Funded by University College London Hospitals National Health Service (NHS) Foundation Trust, The Rosetrees Foundation, the National Institute for Healthcare Research (NIHR) Research for Patient Benefit (RfPB) stream (reference: PB-PG-1216-200113), St Peter’s Charitable Trust, and the Jon Moulton Charity Trust (charity no. 1109891). No funding body have had, nor will have any role in the design, conduct, analysis, interpretation, or dissemination of the study protocol or its final outputs.
Author details {5a}	1. Division of Surgery & Interventional Science, University College London, London, UK. 2. University College Hospital London, Department of Urology, Westmoreland Street Hospital, 16-18 Westmoreland Street, London, W1G 8PH. 3. University College Hospital London, Department of Histopathology, 235 Euston Road, London NW1 2BU. 4. Division of Medicine, University College London, Charles Bell House, 43-45 Foley Street, London, W1W 7JN. 5. Department of Applied Health Research, University College London, 1-19 Torrington Place, London WC1E 7HB. 6. North Bristol Hospitals Trust, Department of Urology, Southmead Hospital, Southmead Lane, Westbury-on-Trym, Bristol, BS10 5NB. 7. North Bristol Hospitals Trust, Department of Histopathology, Southmead Hospital, Southmead Lane, Westbury-on-Trym, Bristol, BS10 5NB. 8. Sheffield Teaching Hospitals NHS Trust, Department of Urology, Royal Hallamshire Hospital, Glossop Road, S10 2JF. 9. Sheffield Teaching Hospitals NHS Trust, Department of Histopathology, Royal Hallamshire Hospital, Glossop Road, S10 2JF.
Name and contact information for the trial sponsor {5b}	University College London (UCL) Sponsor’s reference: 17/0443 Contact name: Ms Suzanne Emerton Address: Maple House, 149 Tottenham Court Rd, Kings Cross, London W1T 7BN
Role of sponsor {5c}	The trial sponsor did not provide any funding for the study. UCL has the role of research governance sponsor of NeuroSAFE PROOF. UCL adopted the study after the Joint Research Office at UCL and University College London Hospitals conducted a trial adoption process that involves reviewing the protocol to ensure conformity to high standards of trial conduct. UCL is responsible for oversight of the study. The sponsor plays no role in data collection, management, analysis and interpretation of data, or dissemination of results.

## Introduction

### Background and rationale {6a}

Prostate cancer (PC) is the second most common non-skin cancer among men [1]. In the United Kingdom (UK), it accounts for nearly 45,000 new diagnoses and 12,000 deaths each year, and incidence is rising [2]. Robotic-assisted radical prostatectomy (RARP) is a first-line curative treatment option for treatment of localized disease. Unfortunately, erectile dysfunction and urinary incontinence are common adverse side effects following RARP that can negatively impact quality of life [3, 4]. These functional results may improve when the neurovascular bundles (NVBs) are preserved during a nerve-sparing (NS) RARP [5, 6].

However, during NS RARP, the dissection planes are closer to the prostate than during non-NS, which poses a risk of positive surgical margins (PSM) and potentially leaving tumour tissue behind. This entails the subsequent need of secondary treatments, increasing patient burden and risk of associated side effects [7].

Therefore, selecting men for NS RARP without jeopardizing the chance of cure by incurring in PSM requires careful planning. Current available planning techniques such as digital rectal examination, transrectal ultrasound [8], multiparametric magnetic resonance imaging (mpMRI) [9], and predictive nomograms [10] are limited by low accuracy and operator dependent variability.

The NeuroSAFE technique is the intra-operative histological frozen section (IFS) analysis of the prostatic postero-lateral prostate margins. It was originally described by the Martini-Klinik in Hamburg, Germany [11]. A recent systematic review performed by our group on IFS during RARP [12] identified three retrospective observational studies suggesting NeuroSAFE may safely increase NS during RARP without increasing PSM or cancer recurrence [13–15]. These studies, and a more recent retrospective cohort study by Fossa et al [16], suggest potential for improving EF preservation using the NeuroSAFE technique. However, all of these studies were at moderate or serious risk of bias [17]. This manuscript describes the protocol for the first multicentre, prospective, randomised controlled trial (RCT) to evaluate the impact of the NeuroSAFE technique on erectile function (EF) of men with PC.

### Objectives {7}

The primary objective of the NeuroSAFE PROOF RCT is to assess whether NeuroSAFE RARP is superior to RARP without NeuroSAFE technique (standard RARP) in preserving EF. Key secondary objectives include to evaluate short term urinary continence recovery, oncological control, quality of life parameters, and health economics between both approaches.

### Trial design {8}

**Fig. 1:**
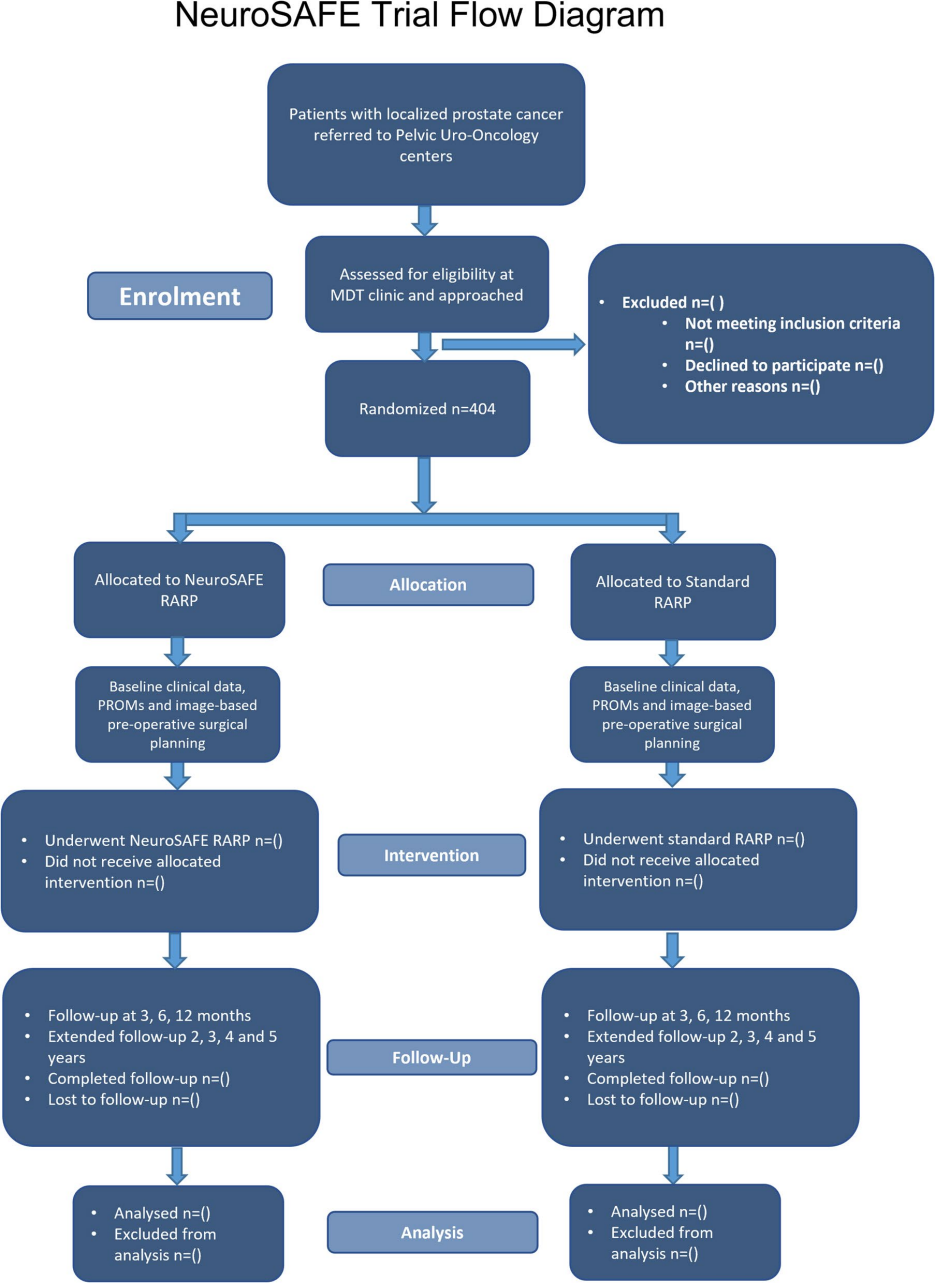
NeuroSAFE PROOF CONSORT patient flow diagram

NeuroSAFE PROOF is a superiority, multi-centre, single-blinded RCT in which men are allocated in a 1:1 ratio to NeuroSAFE RARP or standard RARP (Fig. 1 CONSORT flow diagram). This is the first properly powered trial to randomise men between RP conducted with the NeuroSAFE technique and RP conducted without the NeuroSAFE technique. We have previously published an earlier version of the protocol of the NeuroSAFE PROOF feasibility trial, which demonstrated feasibility to proceed to this definitively powered RCT [18, 19]. In relation to the IDEAL recommendations for the development and evaluation of complex interventions, the NeuroSAFE PROOF RCT most closely aligns with stage 3 (Assessment) [20].

## Methods: participants, interventions, and outcomes

### Study setting {9}

Participants are recruited from regional UK NHS pelvic Uro-Oncology RARP centres. In order to become a NeuroSAFE PROOF trial site, the centre must have identified their interest to the Trial Management Group (TMG), have the expertise to perform the NeuroSAFE technique, and have well-developed RARP programs (i.e. routinely performing at least 200 cases per year and undergoing satisfactory NHS quality assurance and safety visits). There are currently 4 sites recruiting patients: Westmoreland Street Hospital (University College Hospital London (UCLH)), Southmead Hospital (North Bristol Hospitals Trust), Royal Hallamshire Hospital (Sheffield Teaching Hospitals NHS Trust), and Queen Elizabeth University Hospital (NHS Greater Glasgow & Clyde).

### Eligibility criteria {10}

The key eligibility criteria include men with good baseline EF defined as a score > 21 on the International Index of Erectile Function first 5 questions (IIEF-5), who are opting to undergo RARP as primary treatment for localized PC. Eligible patients must have had their case discussed at an NHS cancer multi-disciplinary team meeting and be deemed suitable for RARP before being approached for consenting and randomization. See Tables 1 and 2 for full inclusion and exclusion criteria.

**Table 1:** Inclusion criteria

i. Men opting to undergo RARP for localized PC (including radiological T3a) ii. Potent men (baseline IIEF-5 score 22–25 not using oral medications or erectile aids to improve erection rigidity) iii. Men who are continent of urine (no self-reported urinary incontinence) iv. Ability to read English language sufficiently to understand the participant information sheet (PIS) and respond to trial questionnaires v. Able to give written informed consent to participate

**Table 2:** Exclusion criteria

i. Unfit to undergo RARP ii. Known overactive bladder resulting in urinary incontinence iii. Any prior or current treatment for PC (hormonal, surgical, radiotherapy) iv. NS deemed futile due to locally advanced disease by surgeon and/or radiologist v. Known metastatic PC diagnosed by staging scans vi. Any other contemporary malignancy requiring oncological treatment	

### Who will take informed consent? {26a}

An ethics committee approved patient information sheet (PIS) is provided to potentially eligible participants. Prospective participants are given time to read and understand the PIS before being re-approached to consider enrolment. Written informed consent is obtained from each patient prior to trial entry and the collection of baseline trial assessments (see Supplementary Material S3 for informed consent form (ICF)). The investigator, or their designee, must ensure adequate explanations of the trial whilst reinforcing that participation is voluntary and that they can withdraw at any time without prejudice to his subsequent treatment. Members of staff who are trained to take consent, as indicated by the principal investigator (PI) on the delegation log for that site, take written informed consent in a face-to-face visit. After COVID-19 related disruption, a protocol amendment allowed the use of an electronic remote consent platform that has enabled to continue recruitment complying with recommended governmental advice [21, 22].

### Additional consent provisions for collection and use of participant data and biological specimens {26b}

The NeuroSAFE PROOF team works closely with other PC research teams, including the Molecular Diagnostics and Therapeutics Group at UCL. As such, we invite participants in the NeuroSAFE PROOF trial to consent to the use of their prostatic tissue for further translational cancer biology research. Details of the proposed tissue sampling are described in the approved PIS and are included as an additional ‘opt-in’ on the ICF. A prospective participant may prefer not to consent to tissue sampling for molecular diagnostics and therapeutics and may still consent to being involved in the NeuroSAFE PROOF trial.

### Interventions

#### Explanation for the choice of comparators {6b}

The National Prostate Cancer Audit report 2020 shows that 89% of UK radical prostatectomy is now performed by RARP [23]. RARP is associated with a lower operative blood loss, blood transfusion rate than laparoscopic surgery and a shorter hospital stay than open surgery [24]. RARP results in oncological outcomes which are non-inferior to open or laparoscopic surgery [25, 26]. Current UK NS practice during RARP involves the operating surgeon deciding which nerves he feels he can spare based on the clinical examination, mpMRI, and biopsy findings. This standard of care was selected as our control arm. Prediction of extra-prostatic extension (EPE) of prostate cancer to avoid PSM is paramount. Various techniques have been proposed to predict EPE, but all have their limitations. Transrectal ultrasonography and digital rectal examination (DRE), even in combination, are hampered by insufficient sensitivity (71.1%) and specificity (41.1%) [8]. The use of pre-operative multiparametric magnetic resonance imaging (mpMRI) for prediction of EPE has variable reported diagnostic accuracy [9]. Lastly, EPE prediction nomograms have been widely developed; however, a systematic review and external validation study by Rocco et al. demonstrates their performance is not reliable when applied in centres away from where they originate, and such tools fail to define the side, location, or volume suspicious for EPE [10].

A systematic review has been performed by our group on IFS evaluation of the prostate margin during radical prostatectomy [12]. Ten non-randomized comparative studies (including 16, 897 patients) were retrieved. According to risk of bias assessment, seven studies suffer from serious risk of bias, whereas three studies suffer from moderate risk of bias. Performance of IFS greatly differed technically between studies. Eight studies report a reduction in rates of PSM (− 1.4 to − 14.5%) with the use of IFS and two studies report higher PSM rates (+ 0.4% to + 10%) in the IFS group. Four studies that perform IFS systematically at the posterolateral margin of the prostate (the NeuroSAFE technique) all report either improved NVB preservation or improved EF recovery. Our groups’ conclusions included that, no RCTs were identified, and most of the included studies were at high risk of bias. Furthermore, only very few of the studies included results on either long term oncological or functional outcomes. Therefore, we decided to evaluate NeuroSAFE as our intervention in a randomized prospective study.

#### Intervention description {11a}

##### Robot-assisted radical prostatectomy

The TIDieR checklist for better reporting of interventions accompanies this protocol in the Supplementary Material [27]. Complete description of RARP technique is out of scope of the scope of this report. RARP is carried out using the DaVinci® surgical system (Intuitive Surgical, Sunnyvale, CA, USA) as per standard of care in the NHS and under general anaesthetic. Detailed operation timings are recorded prospectively for all surgeries. Patients are discharged from hospital with an indwelling urethral catheter which is removed in the outpatient department as per standard local practice.

In the NeuroSAFE PROOF study, for all participants, the mpMRI and clinical information (including prostate biopsy results) is used to inform a pre-operative image-based surgical planning meeting. This includes a surgeon and a consultant uro-radiologist with at least 2 years’ experience of interpreting mpMRI. At this meeting, both surgeon and radiologist are blinded to treatment allocation before providing a NS recommendation per side of the prostate that is recorded as (1) Yes, (2) No, or (3) ‘DRE recommended’ [to help guide decision according to surgeon discretion]. Although the pre-operative image-based planning meeting’s aim is to assist the surgeon’s NS decision, the final performance of NS in both arms of the trial is at the discretion of the consultant urological surgeon.

Immediately following RARP the operating surgeon is asked to grade the quality of NS performed on each side in the following [28]:

- *Intrafascial NS*: Lateral pelvic fascia (LPF) is taken just outside the prostate capsule. Represents the greatest possible NS
- *Interfascial NS*: LPF is taken just outside the layer of the veins of the prostate capsule. Still largely preserving the large neural trunks (also known as the NVBs)
- *Limited NS*: Incision through the outer compartment of LPF or dissection plane between interfascial and wide excision (also known as partial, incremental, or semi-NS)
- *Non-NS*: Wide excision of LPF and Denonvilliers’ fascia

##### Control arm: standard RARP

Standard RARP is performed as per NHS routine practice at participating sites. The surgeon’s NS strategy is decided upon using the tumour biopsy information, the pre-operative image-based meeting (described above), and the DRE performed under general anaesthesia by the operating surgeon at the time of RARP.

##### Intervention arm: NeuroSAFE RARP

NeuroSAFE RARP is performed in accordance with the methods described in our feasibility study [18, 19]. Further information about the steps of the NeuroSAFE RARP can be viewed in the video prepared by the TMG [29]. Briefly, unless it is technically impossible or deemed to be unsafe because of intra-operative findings, the surgeon starts with a bilateral NS RARP, after which the prostate is removed from the body through an enlarged umbilical incision as soon as it has been detached from surrounding structures. Upon removal from the patient, the safety of the NS is checked by performing IFS analysis of neurovascular structure adjacent margin (the NeuroSAFE technique). Whilst the NeuroSAFE technique is being performed, pneumoperitoneum is re-established with the Alexis Laparoscopic System (Applied Medical Rancho Santa Margarita, CA) and the final steps of the RARP, such as the urethrovesical anastomosis and pelvic lymphadenectomy (where appropriate) may be performed. Directly following RARP, the operating surgeon is asked to grade the quality of NS performed on each side as above. Based on analysis of the results of the NeuroSAFE PROOF Feasibility Study, the trial team estimated the additional cost of the NeuroSAFE technique during RARP, including additional time spent in the operating room (OR), to be £625.10 (Supplementary Material S1 for further detail).

##### NeuroSAFE technique

**Fig. 2:**
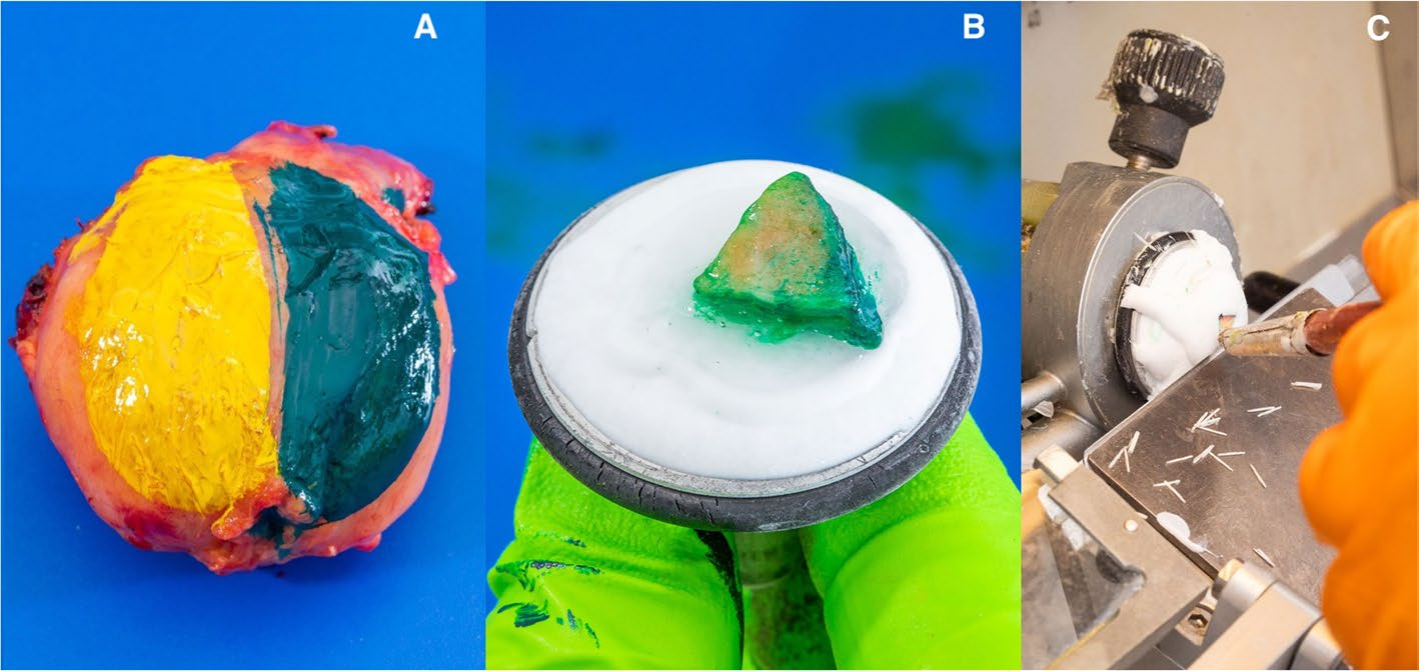
Images showing the performance of intra-operative frozen section as per the NeuroSAFE technique. **A** Ink stains the left (yellow) and right (green) neurovascular structure adjacent prostate margin, respectively. **B** After cleaving the right side and slicing perpendicularly a 5-mm piece of prostate tissue sits on the cryostat before freezing. **C** Once embedded in optimal cutting temperature compound and frozen, 5-μm sections are prepared on the microtome before staining

Immediately upon removal of the prostate, the postero-lateral neuro-vascular structure adjacent surfaces are painted in theatres by the operating surgeon (Fig. 2A). The whole prostate is then transported to the histopathology department where the entire inked margin is cleaved by a straight blade. This inked postero-lateral portion of the prostate is further divided by perpendicular cuts at intervals of 4–5 mm from apex to base. Depending on the size of the prostate, a minimum of 4 and a maximum of 7 pieces are obtained. Each piece of prostate tissue is then embedded into optimal cutting temperature compound on a cryostamp and frozen (Fig. 2B). The frozen prostate tissue is then transferred to the cryostat for sectioning at a tissue thickness of 5 μm (Fig. 2C) before staining with haematoxylin and eosin. Slides are examined by a consultant genito-urinary histopathologist and the results are conveyed to the console surgeon in the OR.

##### NeuroSAFE IFS reporting

The results from the serial sections are reported in relation to laterality and distance from prostate apex. When there are no cancer cells touching the inked margin on an individual section, the slide is reported as clear/negative. When all sections of a side are clear/negative, the margin is considered negative (NSM) and no further tissue resection is performed on that side. When the margin is positive, further information is collected to inform the intra-operative secondary resection (SR) decision including grade and length of cancer seen at the margin. Once the reporting pathologist has given a frozen section margin assessment (Supplementary Material S2 for model reporting proforma), each piece of prostate tissue is defrosted in warm water and placed individually in formalin for paraffin embedding and subsequent concordance assessment. All prostatectomy specimens are processed in accordance with the standard procedures recommended by the International Society of Urologic Pathology [30]. Final pathological reports are recorded prospectively for analysis.

##### SR indication

**Fig. 3:**
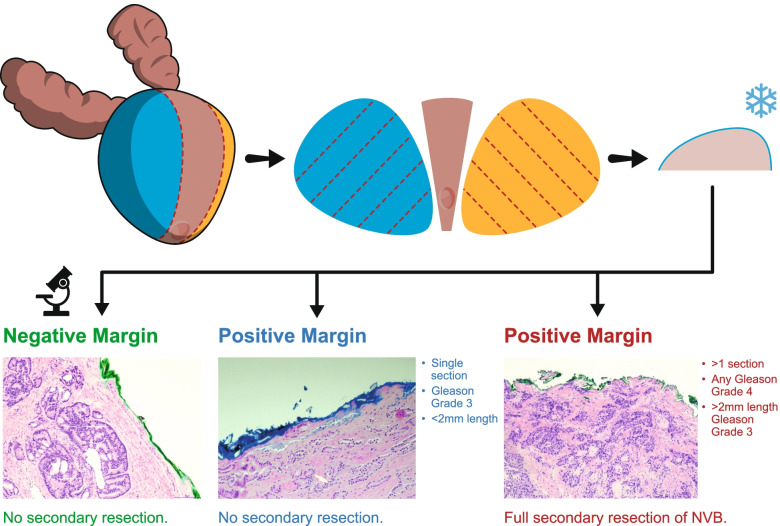
The images depict cut up of prostate during the NeuroSAFE technique and the intra-operative surgical response (i.e. secondary resection (SR) or no SR) according to the margin status

When NSM is reported by the NeuroSAFE technique, this results in the preservation of the corresponding original ipsilateral NS status, and the operation is completed in the standard fashion. SR is indicated in the presence of a PSM with any of the following features (see Fig. 3):

- When more than 1 positive section is found on a side (any grade or length of cancer),
- Any Gleason grade group 4 pattern or above at the inked surgical margin,
- More than 2 mm Gleason grade 3 pattern present at a single section either in continunity or cumulatively.

This protocol (discussed in the ‘Discussion’ section) has been developed in line with our published experience from the NeuroSAFE PROOF Feasibility Study [19] and based on the experience of other centres performing IFS.

##### SR technique

When the NeuroSAFE technique PSM prompts SR, a full excision of the entire ipsilateral NVBs is attempted. Thus, all tissue from the cut edge of Denonvilliers’ fascia medially, the pararectal fat laterally, the bladder cranially, and just beyond the urethrovesical anastomosis (including the puboprostatic ligament and Walsh’s pillar) caudally is removed en bloc by the surgeon as originally described by Schlomm and colleagues [13]. SR tissue is sent for routine formalin-fixed and paraffin-embedded histological analysis and is not analysed intra-operatively. When SR is performed on one or any side, this is prospectively recorded as non-NS in the operation note.

### Criteria for discontinuing or modifying allocated interventions {11b}

If the surgeon finds it is technically impossible or deemed to be unsafe to perform NS in any of the two arms due to intra-operative findings, the operation will be carried out as a non-NS procedure. Such cases are recorded prospectively for descriptive presentation.

### Strategies to improve adherence to interventions {11c}

Surgeon heterogeneity significantly affects functional and oncological outcomes after RARP [31]. To minimize this potential source of confounding, surgeons and surgical teams participating in NeuroSAFE PROOF will require accreditation from the TMG. Individual surgeons performing RARP (both intervention and control) will have completed more than 100 cases prior to involvement in the study and have submitted these data to the British Association Urological Surgeons Oncology database. Participating surgeons must have visited the central site (UCLH) before their site initiation visit (SIV) to receive teaching and standardization in the surgical and histopathological aspects of NeuroSAFE RARP. Subsequently, researchers from the central site (including at least the chief investigator (CI) and a study histopathologist) must visit the site for the first NeuroSAFE RARP performed, to help with the standardization of the NeuroSAFE technique in accordance with trial protocol.

### Relevant concomitant care permitted or prohibited during the trial {11d}

There is no difference in participant follow-up according to treatment arm (see Table 3 and Fig. 1 CONSORT Flow Diagram). Management of Foley catheters and the first appointment after RARP is arranged by local NHS clinicians at 2 and 6 weeks, respectively. Following this, at 3 months (visit 2), 6 months (visit 3), 12 months (visit 4), and 24 months (visit 5), the research team conduct follow-up appointments liaising with local clinicians where necessary. At each visit, a prostate specific antigen test (PSA) is requested. Biochemical recurrence (BCR) is defined as PSA > 0.2 ng/mL, and biochemical failure is defined as a PSA that never falls below 0.2ng/mL. As per the report from the RADICALS-RT and RAVES trials on the timing of radiotherapy after RP, salvage treatments should not be initiated before BCR [32]. All adjuvant treatments and PSA results are prospectively recorded onto the trial database. Participants undergo post-operative EF recovery programs according to local routine practice, and there will be no protocolized/structured penile rehabilitation program as this may limit external validity of the study.

**Table 3:** SPIRIT figure delineating participant timeline in NeuroSAFE PROOF RCT

	Study period									
	Enrolment	Allocation	Post-allocation							
			Visit 1 treatment	Visit 2 (3 months*)	Visit 3 (6 months*)	Visit 4 (12 months*)	Visit 5 (2 years*)	Visit 6 (3 years*)	Visit 7 (4 years*)	Visit 8 (5 years*)
*Enrolment* PSA and PCa biopsy results (according to routine PCa diagnostic pathway)	x									
Eligibility screen	x									
Informed consent	x									
Randomization		x								
*Interventions* Image-based (mpMRI) pre-operative surgical planning conference		x								
Standard RARP (control)/NeuroSAFE RARP (intervention)			x							
*Assessments* Peri-op adverse events			x	x						
PROMs (IIEF-15, ICIQ, EQ-5D-5L, Rand-36)	x			x	x	x	x			
PSA				x	x	x	x	x	x	x
Adjuvant treatments assessment				x	x	x	x	x	x	x
Health resource diaries				x	x	x	x			

*± 6 weeks

### Provisions for post-trial care {30}

Participants will continue standard of care oncologic follow-up in their original NHS referring centres after 2 years of follow-up at the surgery performing site. Subsequent yearly study visits will be conducted by phone and reviewing electronic health records for study purposes, but findings will not impact clinical care.

UCL holds insurance against claims from participants for injury caused by their participation in the trial. Participants may be able to claim compensation if they can prove that UCL has been negligent. However, as this trial is being carried out in a hospital, the hospital continues to have a duty of care to the participant of the trial. UCL does not accept liability for any breach in the hospital’s duty of care or any negligence on the part of hospital employees. This applies whether the hospital is an NHS Trust or otherwise. Participants may also be able to claim compensation for injury caused by participation in this trial without the need to prove negligence on the part of UCL or another party. Participants who sustain injury and wish to make a claim for compensation should do so in writing in the first instance to the CI, who will pass the claim to the sponsor’s insurers, via the sponsor’s office.

### Outcomes {12}

#### Primary outcome

Comparison of the proportion of men who achieve EF recovery at 12 months in the intervention (NeuroSAFE RARP) compared to control (standard RARP) arm.

#### Primary endpoint

Preservation of EF is defined as a score of ≥ 21 on the IIEF-5 at 12 months after treatment (visit 4).

Men are permitted to consider their answers to the IIEF-5 questions with or without the use of oral erectile medications such as phosphodiesterase-5 inhibitors (PDE5is), as is routine in the post-RP literature [14]. However, the use of erectile aids (such as the vacuum pump device, intra-cavernosal injections and penile prostheses) is not permitted.

#### Secondary outcomes

- Secondary outcome 1: Urinary continence. Comparison of the proportion of men in each arm who achieve continence at 3 months following surgery measured using the validated International Consultation of Incontinence Questionnaire (ICIQ) short form, where continent is defined as a score of 5 or less on the sum of items 3, 4, and 5 [28]. In recognition that alternative thresholds can be used for defining continence after RP using the ICIQ, continence recovery will be reported in using several definitions including those described in the MASTER trial [33].
- Secondary outcome 2: BCR. Comparison of the proportion of men in each arm who develop PSA > 0.2 ng/mL within 12 months post-surgery.
- Secondary outcome 3: Additional oncological treatments. Comparison of the proportion of men who receive adjuvant or salvage treatment. Men who receive additional cancer treatment without having reached a PSA threshold of 0.2 ng/ml after RARP will be considered to have undergone ‘adjuvant treatment.’ Men who receive additional cancer treatment following a PSA that rises above 0.2 ng/ml will be considered receiving salvage treatment but will be primarily considered as having had BCR. The trial team will make efforts to minimize administration of adjuvant treatment as per recent evidence [25] and to minimize confounding within the oncological follow-up of patients. Reasons for all further oncological treatment will be documented and tabulated where possible.
- Secondary outcome 4: Quality of life. Comparison of mean quality-of-life scores according to treatment arm using the EQ-5D-5L and RAND-36 patient-reported outcome measures (PROMs) at 12 months.
- Secondary outcome 5: PSM. Descriptive tabulation of PSM rates between the treatment arms according to NSM, length of PSM and whether PSM was intra- or extra-prostatic.
- Secondary outcome 6: Health economic analysis. Descriptive analysis using patient health resource diaries to compare healthcare resource use and cost according to treatment arm.
- Secondary outcome 7: EF recovery at 2 years. Proportion of men achieving preservation of EF, using the same definition as the primary endpoint, according to treatment arm.

#### Participant timeline {13}

See Table 3 and Fig. 1 CONSORT patient flow diagram.

#### Sample size {14}

Sample size calculation was based on the results of the NeuroSAFE PROOF feasibility study data. Patients from the feasibility study will not be included in the main trial analysis. A sample of 416 patients (374 evaluable) is needed to detect a 14% or higher difference between groups with 90% power, alpha of 0.05, and a loss to follow-up rate of 10%. EF preservation rates from the feasibility study have not been presented here to protect equipoise and because the feasibility study was not powered to detect treatment effect estimates. The Independent Data Monitoring Committee (IDMC) reviewed the sample size assumptions on 16 February 2021, unblinded to results from the first 150 men recruited to the main trial and advised the study to continue recruitment to the original number of 404 men (364 evaluable men). The IDMC has continued reviewing the recruitment target during the trial.

#### Recruitment {15}

The NeuroSAFE PROOF recruits patients attending the outpatient departments of participating NHS pelvic uro-oncology RARP centres. The clinical teams managing patients who are electing to have RARP identify potential trial participants to the trial team. Recruitment over the past 12 months has been severely disrupted by the coronavirus disease 2019 (COVID-19) pandemic; however, the trial has met its halfway point for its recruitment target during this time.

### Assignment of interventions: allocation

#### Sequence generation {16a}

Patients are randomised using an online system by designated study personnel at each site in a 1:1 ratio to either NeuroSAFE RARP or standard RARP immediately after recruitment [34]. A computer-generated adaptive minimization algorithm that incorporates a random element is used to ensure treatment groups are balanced (stratified) for centre.

#### Concealment mechanism {16b}

Once participants sign the informed consent and eligibility criteria is verified the use of the centrally based computer algorithm prevents manipulation or prediction of allocation by trial personnel, all entries are recorded, and no further modification is possible.

#### Implementation {16c}

Potential candidates are identified by designated urology consultants, research nurses, or research fellows at each participant centre. After enrolment is confirmed, the same trial personnel access the electronic randomization platform through any electronic device with internet connectivity, randomization is performed, and allocation registered in the database.

### Assignment of interventions: blinding

#### Who will be blinded {17a}

Participants will be blinded to their treatment allocation. Following feedback received by the NeuroSAFE PROOF trial team during a PPI workshop in September 2018, participants are informed of the NS status following surgery by the clinical team. They are not, however, informed of their treatment allocation to minimize bias with reporting their functional outcomes.

Radiologist and surgeons are blinded to treatment allocation during the pre-operative image-based surgical planning meeting. Subsequent blinding of clinicians is largely unfeasible as each site trial team is involved in the logistics of arranging the NeuroSAFE technique (or not) for participants. The study statistician will be blinded to the allocation until the end of the analysis.

#### Procedure for unblinding if needed {17b}

Participants will be unblinded following 5 years of follow-up, if requested. The study statistician will be blinded to the allocation until the end of the analysis.

### Data collection and management

#### Plans for assessment and collection of outcomes {18a}

Validated and disease-specific quality of life PROMs IIEF-15 and IIEF-5 for EF recovery, ICIQ for urinary continence, and EQ-5D-5L and RAND-36 for overall health-related quality of life are completed by all participants to evaluate all aspects of recovery.

Time points (see Fig. 1 CONSORT flow diagram):

i. Baseline/preoperative: directly after consent
ii. Outpatient follow-up visits. The first follow-up appointment used for collection of PROMs as per the trial protocol occurs 3 months after treatment (visit 2). Further outpatient follow-up visits for PROMs collection occur at 6 months (visit 3), 12 months (visit 4), and 24 months (visit 5). After visit 5, patient record data and telephone follow-up are used to ascertain oncological outcomes until 5 years post-treatment

Beyond 2 years after surgery, there is limited potential change in EF and continence scores. Therefore, annual subsequent visits will be limited to oncological surveillance.

#### Plans to promote participant retention and complete follow-up {18b}

Once a participant is enrolled or randomised, the local site will make every reasonable effort to follow the patient for the entire study period. It is estimated that loss to follow-up/withdrawal after treatment will be no more than 10%. Study site staff are responsible for developing and implementing local standard operating procedures. Prior to March 2020, all trial follow-up visits were performed face to face; however, since the start of the COVID-19 pandemic and restrictions on movement, all follow-up visits have been conducted over the telephone and patients have sent their PROMs questionnaires to the local trial centre by post with participants called to the hospital only where necessary. In case a participant misses a trial related visit, trial personnel reach out to the patient by post or phone. Questionnaire responses are considered valid +- 6 weeks from the official date of the visit.

#### Data management {19}

Patients’ PROMs and clinical information are added to an electronic database by a responsible data manager for each site; further information is available in the supplementary material.

#### Confidentiality {27}

Data is handled according to regulatory requirements and be protected according to EU Law Enforcement Directive EU2016/680 (which is now incorporated into UK GDPR) and the Data Protection Act 2018 as well as local data protection requirements.

#### Plans for collection, laboratory evaluation, and storage of biological specimens for genetic or molecular analysis in this trial/future use {33}

Prostate tissue from the patients who consent to biobanking is reserved for collection by the Molecular Diagnostics and Therapeutics Group without compromising the performance of the NeuroSAFE technique, the assessment of the prostate specimen for final histological diagnosis, nor elongating the length of the operation/time a man spends under general anaesthetic. The performance of fresh tissue collection from the prostates of men involved does not influence decisions about their clinical care. Methodology of these investigations includes but is not limited to ex vivo cultures, genomics, and immunohistochemistry. All samples are pseudonymized, and all data is stored securely within NHS frameworks. All tissue is stored and tracked in accordance with the Human Tissue Act, with regular internal audits to ensure sample and data security.

### Statistical methods

#### Statistical methods for primary and secondary outcomes {20a}

A formal statistical analysis plan (SAP) will be finalised before a database lock and any analysis undertaken thereafter. This SAP will be published as an additional file when available in *Trials* journal. All analyses and data summaries will be conducted on a modified intention to treat basis, where those who did not undergo the allocated treatment will be excluded.

#### Interim analyses {21b}

No interim analysis is planned during the conduct of the trial though the IDMC reviews safety data, including oncological outcomes, at each quarterly meeting.

#### Methods for additional analyses (e.g. subgroup analyses) {20b}

Additional subgroup analysis of the primary outcome will be performed on men who did not receive a pre-operative radiologist recommendation for bilateral nerve sparing. Additional subgroup analysis: patients who did not receive adjuvant treatment (adjuvant therapy defined as any additional treatment without a PSA > 0.2 ng/ml).

- Sensitivity analyses of the primary outcome will be performed at 12 months using 
  - IIEF-5 score using a threshold ≥ 15
  - IIEF-5 item 3 score only using a threshold of ≥ 2
- Descriptive analysis of IIEF-5 at 3, 6, 12, and 24 months post-surgery

#### Methods in analysis to handle protocol non-adherence and any statistical methods to handle missing data {20c}

Multiple imputations will be used to handle missing data using chained equations in STATA® (StataCorp). The primary outcome will be based on imputed analysis to take advantage of the availability of the IIEF-5 and ICIQ outcomes across multiple time points within patients. This is particularly relevant because of COVID-19 disruptions to patient outcome data returns. Sensitivity analyses will be reported based on (i) complete data only (ii) best-case results and (iii) worst-case results. For continence outcomes, ICIQ scores of zero will be carried forward to following time points.

#### Plans to give access to the full protocol, participant level-data, and statistical code {31c}

On completion of the study, the results will be disseminated to patients through their local site, and all participants will be invited to an online PPI results presentation. The results will be published following peer review, and anonymised data will be presented at national and international conferences. No plans for dissemination of the full protocol, data, or statistical code are contemplated.

### Oversight and monitoring

#### Composition of the coordinating centre and trial steering committee {5d}

The NeuroSAFE PROOF TMG consists of the CI, PIs, the trial manager, representatives of trials units, site staff including trial coordinators and involved clinicians, and database managers. This group is responsible for:

- Study planning,
- Preparation of protocols and revisions,
- Assistance with local ethical committee permissions,
- Preparation of investigators brochure and CRFs,
- Organization of the TSC,
- Preparation of progress reports,
- Preparation of reports to funders,
- Reporting of adverse events and serious adverse events (SAEs) to appropriate bodies where necessary,
- Trial master file,
- Budgets and contracts,
- Advice for PIs,
- Site initiations visits,
- Data management including completeness checks,
- Randomization services,
- Maintenance of trial systems
- Publication of study reports.

The Trial Steering Committee (TSC) consists of a team independent of the NeuroSAFE PROOF TMG and independent of the PIs. It comprises independent chairs, advisory board members, and chief investigators. TSC’s role is to monitor and supervise the progress of the study, consider the recommendations of the IDMC, and make recommendations on future study conduct.

#### Composition of the data monitoring committee, its role and reporting structure {21a}

The IDMC consists of an independent chair, clinical representative, patient representative, and statistician. The role of the IDMC is to safeguard the interests of trial participants and monitor the overall conduct of the trial through a quarterly review of the oncological outcomes reported in NeuroSAFE PROOF to ensure participant safety.

#### Adverse event reporting and harms {22}

All adverse events are monitored and recorded prospectively. Further information is available in the supplementary materials.

#### Frequency and plans for auditing trial conduct {23}

The IDMC reviews oncological outcome data at regular intervals (3–4 months) and will advise the TSC and the CI if there is evidence that oncological outcome is being compromised in a group of patients.

#### Plans for communicating important protocol amendments to relevant parties (e.g. trial participants, ethical committees) {25}

Protocol amendments are disseminated to relevant parties by the trial manager. All versions have been approved by the sponsor, TSC, funding bodies, and ethics committee. Current version 5.0 is considered final, and no substantive changes are planned.

#### Dissemination plans {31a}

Unblinded results of the study will be published in peer-reviewed publications and will be presented at relevant national and international conferences. The TMG will work with a patient panel to develop lay reports to disseminate research findings to patient groups and the clinical teams at participating sites.

## Discussion

### Key strengths and limitations

- *Strength*: This is the first RCT to compare NeuroSAFE RARP against RARP performed without the NeuroSAFE technique.
- *Strength*: Using PROMs for primary and secondary outcome analysis puts patients at the centre of the trial design.
- *Strength*: The multi-centre trial design contributes to external validity and will help inform clinical practice in the UK and around the world.
- *Strength*: Detailed prospective collection of BCR rates will help define safety of the NeuroSAFE technique.
- *Strength*: Blinding patients will reduce response and ascertainment bias, but it was not possible to blind all evaluators and clinical teams to treatment allocation.
- *Limitation*: Variability in the NS decision, technical performance of surgery (RARP) between centres and surgeons, and yet unknown adverse features of PC biology that differentially affect oncological outcomes could be potential sources of confounding.

### Regarding EF recovery

The definition of ‘preservation of EF’ after RP is not standardized [5, 35]. Different definitions of EF preservation accounts for some of the wide variations in recovery rates reported in the literature [36]. In this study, the IIEF-5 PROM questionnaire will be used to evaluate EF recovery [37], and a threshold for recovery has been chosen in conjunction with consultation from public and patient involvement (PPI) workshops (see the ‘Public and patient involvement (PPI)’ section) and in accordance with previous authors [38].

### Regarding decision for SR during NeuroSAFE technique

As highlighted above, our trial protocol for the performance of SR (Fig. 3) has been developed in line with our experience from the NeuroSAFE PROOF Feasibility Study [19] and based on the experience of other centres performing IFS. Van der Slot and colleagues in 491 patients found that in no patients with < 1 mm of Gleason pattern 3 at the margin was adenocarcinoma seen in the SR specimen [39]. Conversely, Choi et al. found that Gleason grade pattern 4 and over on at the margin on IFS was predictive of BCR [40]. Several studies regarding PSM on final prostatectomy specimen (as opposed to IFS) have found that men with ≤ 3 mm PSM length had similar BCR-free survival as compared to those with NSM [41, 42].

### Public and patient involvement (PPI)

PPI has been central to the design of the NeuroSAFE PROOF trial at key stages of development. During the feasibility study, feedback received changed the frequency of follow-up from 4 visits during the first 12 months after surgery to 3 and informed the study design on blinding of patients (see Blinding section). More recently, in September 2020, the investigators convened an online PPI session attended by > 40 participants [43]. The audience were consulted in depth using teleconference polling techniques about the definition of EF preservation to select a primary endpoint that would be most meaningful to patients such as those enrolled in NeuroSAFE PROOF. Men who attended this event encouraged the NeuroSAFE PROOF researchers to elect for an IIEF-5 threshold of at least 20 for the definition of EF preservation. Furthermore, PPI representatives are on the Trial Steering Committee (TSC) and the IDMC for NeuroSAFE PROOF and will have oversight of the management of the research analysis.

## Trial status

The NeuroSAFE PROOF Feasibility Study opened recruitment in October 2017 and finished recruiting in December 2018. The definitive NeuroSAFE PROOF study opened to recruitment in January 2019 using the same trial identifier following a substantial amendment to the trial protocol. The trial team decided to transition to the full study through a substantial amendment (i.e. using the same trial identifiers) because it was felt, at the time, that this would be the most expeditious route and would facilitate ongoing conduct of and recruitment to the study at a time when there was considerable momentum. This decision was supported by the trial funder. In this manuscript, we report version 5.0 (May 2021) of the trial protocol. Earlier versions (including the feasibility study protocol) can be accessed on the NeuroSAFE PROOF ClinicalTrials.gov page via complete list of historical versions. All amendments were reviewed and approved by Sponsor, Health Research Authority and the Research Ethics Committee (REC) prior to any research activity permitted within said amendment. Permission for the full NeuroSAFE PROOF to continue to its definitive sample size, including patients recruited under versions 2.0, 3.0, and 4.0, was granted on 16 June 2021 under version 5.0. COVID-19 travel restrictions have prompted the trial teams to conduct follow-up by telephone and collect PROMs by post, telephone, or electronically. At the time of writing, 230 patients have been recruited and randomised to the NeuroSAFE PROOF RCT. Estimated date for recruitment completion is December 2022.

NeuroSAFE PROOF will continue to recruit until the 404th patient is consented, randomised, and undergoes planned intervention. NeuroSAFE PROOF will therefore close when the last participant to undergo treatment completes the 5-year follow-up as per protocol.

## Supplementary Information

**Additional file 1: Supplementary Material S1.** Cost analysis of NeuroSAFE technique demonstrating additional cost total of the procedure in the United Kingdom NHS setting according to the results of the Feasibility Study. **Supplementary Material S2.** Model Intraoperative Frozen Section Reporting Proforma. **Supplementary Material S3.** Informed Consent Form.

## Data Availability

NeuroSAFE PROOF will comply with all information governance and confidentiality guidelines for the performance of clinical research. After completion of the study, the database will be retained on the servers of UCL for ongoing analysis of secondary outcomes. The identification, screening, and enrolment logs, linking participant identifiable data to the pseudo-anonymised subject numbers will be held in written form in a locked filing cabinet. After completion of the study, sites will store screening and enrolment logs securely for 10 years. Access will only be granted to approved research personnel.

## Abbreviations

BCR: Biochemical recurrence
CI: Chief investigator
CIV: Cite initiation visit
CONSORT: Consolidated Standards of Reporting Trials
COVID-19: Coronavirus disease 2019
IDMC: Independent Data Monitoring Committee
DRE: Digital rectal examination
EF: Erectile function
EPE: Extra-prostatic extension
ICIQ: International Consultation on Incontinence Questionnaire
ICF: Informed consent form
IFS: Intra-operative frozen section
IIEF-5: International Index of Erectile Function
LPF: Lateral prostatic fascia
mpMRI: Multi-parametric magnetic resonance imaging
NHS: National Health Service
NIHR: National Institute of Health Research
NS: Nerve sparing
NVBs: Neurovascular bundles
NSM: Negative surgical margin
OR: Operating room
PC: Prostate cancer
PDE5i: Phosphodiesterase type 5 inhibitor
PI: Principal investigator
PIS: Participant information sheet
PPI: Patient and public involvement
PROMs: Patient-reported outcome measure
PSA: Prostate-specific antigen
PSM: Positive surgical margin
RARP: Robotic-assisted radical prostatectomy
RCT: Randomised control trial
REC: Research Ethics Committee
RfPB: Research for Patient Benefit
SAP: Statistical analysis plan
SAEs: Serious adverse events
SIV: Site initiation visit
SR: Secondary resection
TSC: Trial Steering Committee
TMG: Trial Management Group
UCL: University College London
UCLH: University of London College Hospitals
UK: United Kingdom
