# Supplementary material for: NeuroSAFE PROOF: study protocol for a single-blinded, IDEAL stage 3, multi-centre, randomised controlled trial of NeuroSAFE robotic-assisted radical prostatectomy versus standard robotic-assisted radical prostatectomy in men with localized prostate cancer
Source: Trials. 2022 Jul 22;23:584. doi: 10.1186/s13063-022-06421-7 (PMC9306247; doi:10.1186/s13063-022-06421-7)
Supplement: Supplementary file 1 — Additional file 1: Supplementary Material S1. Cost analysis of NeuroSAFE technique demonstrating additional cost total of the procedure in the United Kingdom NHS setting according to the results of the Feasibility Study. Supplementary Material S2. Model Intraoperative Frozen Section Reporting Proforma. Supplementary Material S3. Informed Consent Form. [file 13063_2022_6421_MOESM1_ESM.docx]

**A single-blinded, IDEAL stage 3, multi-centre, randomised controlled trial of NeuroSAFE robotic-assisted radical prostatectomy versus standard robotic-assisted radical prostatectomy in men with localized prostate cancer (NeuroSAFE PROOF): a study protocol.**

**Supplementary Material**

**Data Management**

To ensure high-quality trial conduct, data management will be carried out according to the principles of the International Council of Harmonisation Good Clinical Practice. Data will be entered onto a purpose-designed web-based electronic case report form (eCRF) system. Quality control is carried out routinely. Data types, entries and permitted ranges for answers to every question on the eCRFs are restricted on this web-based system. Automatic validation checks and automatic queries are raised by the system immediately to individual sites in the case of queries. Authorised individuals from the TMG may also check the data for quality and may pose manual queries to site. A proportion of the participating sites’ mpMRI and histopathology may be verified centrally.

NeuroSAFE PROOF will comply with all information governance and confidentiality guidelines for the performance of clinical research. After completion of the study, the database will be retained on the servers of University College London for ongoing analysis of secondary outcomes. The identification, screening and enrolment logs, linking participant identifiable data to the pseudo-anonymised subject numbers will be held in written form in a locked filing cabinet. After completion of the study, sites will store screening and enrolment logs securely for 10 years.

**Safety & Harms**

In addition to routine oncological outcome assessment by the IDMC, the number of adverse events and serious adverse events (SAEs) will be summarised for review by the IDMC. RARP is a major surgery that has several recognized complications and a very low risk of death (less than 1 in 1000). The TMG will ask sites to submit complication data blinded by treatment arm. This will be assigned Clavien-Dindo classification centrally.

All SAEs will be recorded in the medical records, the eCRF, the sponsor’s AE log, and an SAE form. The site principal investigator (PI) or designated individual will complete an SAE form, and the form will be sent to the trial manager within five working days of becoming aware of the event. The CI or site PI will respond to any SAE queries raised by the sponsor as soon as possible. Where the event is unexpected and thought to be related to the procedure, this must be reported by the PI to the trial unit, who will then inform the Health Research Authority within 15 days. The following are expected AEs following RARP with their corresponding likelihoods:

*Intra-operative*

Bleeding (requiring transfusion) - 1%

Visceral injury requiring laparotomy – 1%

Vascular injury requiring laparotomy – 1%

Cardiac event (Myocardial infarction 0.1%, Atrial Fibrillation 1.6%, syncope 1.2%).

*Early post-operative*

Wound related problems; infection (2%), incisional hernia (2%)

Thromboembolic event (deep vein thrombosis or pulmonary embolus 0.8%) Lymphoedema – 1% (higher incidence when lymph node dissection performed)

Anaesthetic problems requiring admission to intensive care unit (2%)

Gastrointestinal – ileus or damage to bowel requiring temporary colostomy.

Seroma – 1%

Urethral Stricture – 2%

*Longer term post-operative*

Urinary incontinence – (temporary) 100%

Erectile dysfunction – (up to 100%)

Long term urine leak – 10%

Adjuvant therapies (including radiotherapy and ADT) – 30%

**Supplementary Material S1.** Cost analysis of NeuroSAFE technique demonstrating additional cost total of the procedure in the United Kingdom NHS setting according to the results of the Feasibility Study.

**Supplementary Material S2.** Model Intra-operative Frozen Section Reporting Proforma.

**Supplementary Material S3.** Informed Consent Form.

**
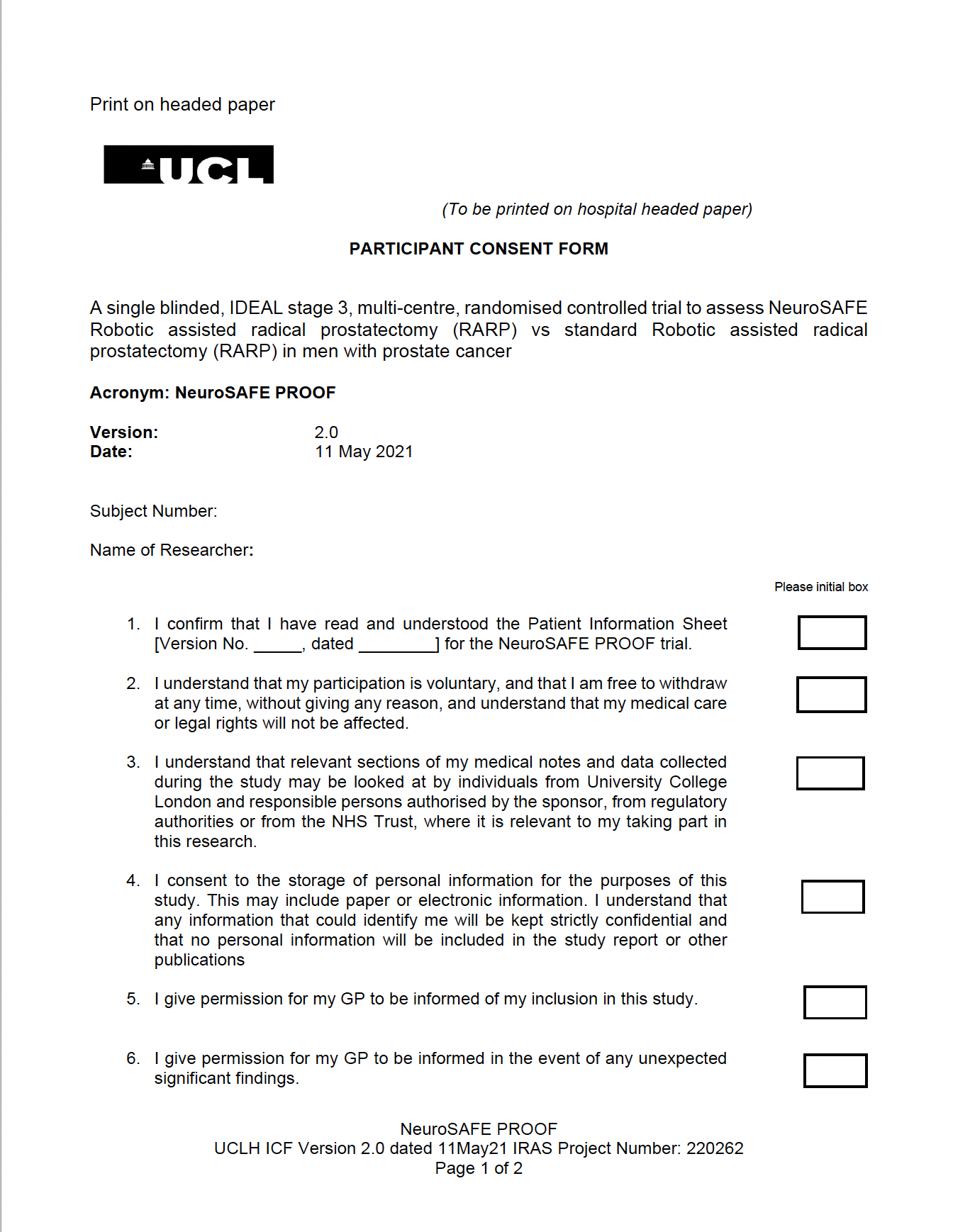
**

**
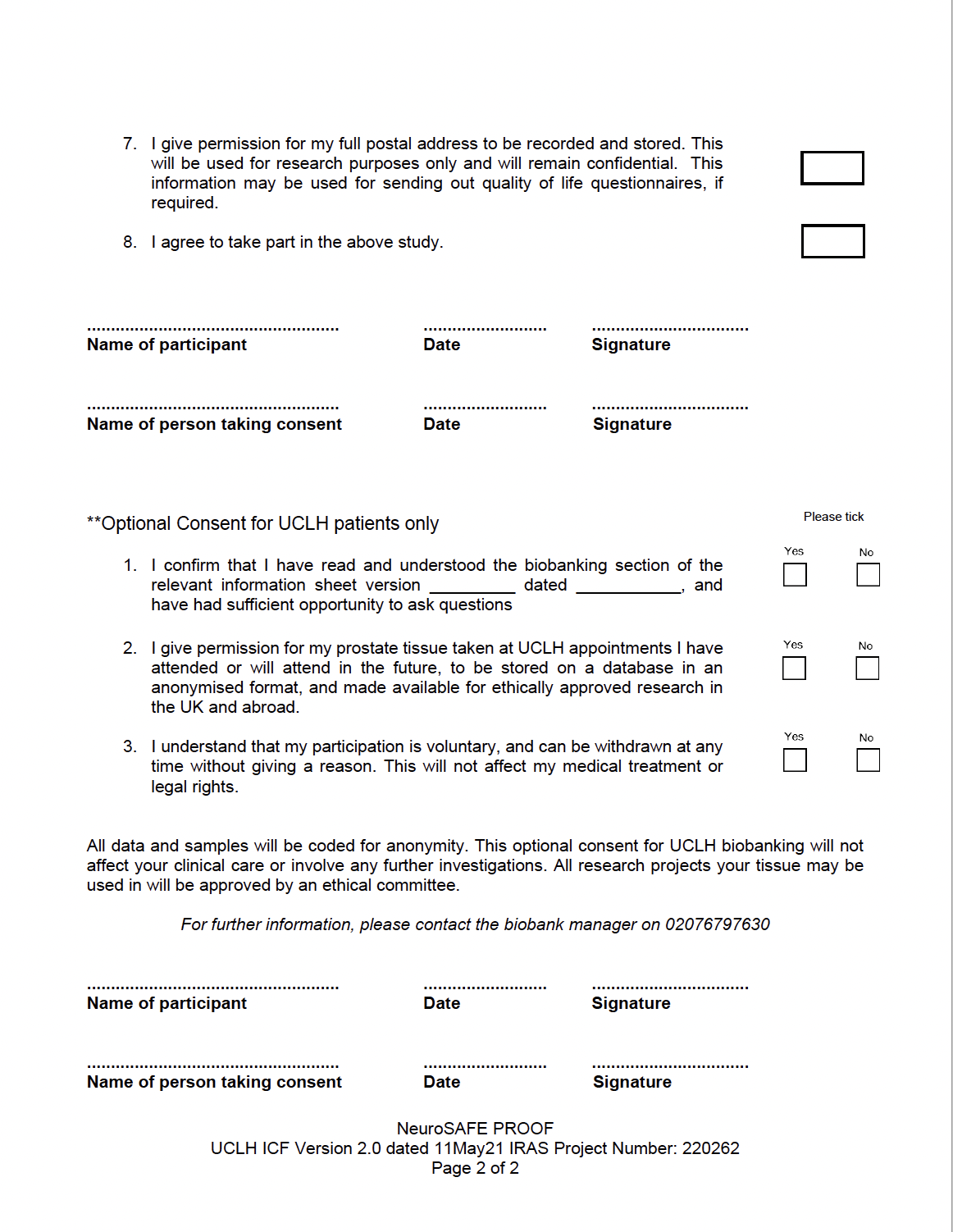
**
